# Identification of bladder cancer subtypes and predictive model for prognosis, immune features, and immunotherapy based on neutrophil extracellular trap-related genes

**DOI:** 10.1038/s41598-023-47824-z

**Published:** 2023-11-27

**Authors:** Changhong Guo, Peiying Li, Xingkui Guo, Xinfen Wang, Bo Liu, Liang Cui

**Affiliations:** 1 Department of Urology, Civil Aviation General Hospital, Beijing, China; 2 Department of Urology, The Fifth Medical Center of the General Hospital of the People’s Liberation Army of China, Beijing, China; 3 Department of Urology, The Second People’s Hospital of Juancheng County, Shandong, China; 4 Department of Urology, The First People’s Hospital of Juancheng County, Shandong, China

**Keywords:** Cancer genetics, Cancer therapy

## Abstract

Bladder cancer is the most common malignant tumor of urinary system, and its morbidity and mortality are increasing rapidly. Although great advances have been made in medical technology in recent years, there is still a lack of effective prognostic and therapeutic methods for bladder cancer. NETs are reticulated DNA structures decorated with various protein substances released extracellularly by neutrophils stimulated by strong signals. Recently, it has been found that NETs are closely related to the growth, metastasis and drug resistance of many types of cancers. However, up to now, the research on the relationship between NETs and bladder cancer is still not enough. In this study, we aimed to conduct a comprehensive analysis of NRGs in bladder cancer tissues to evaluate the relationship between NRGs and prognosis prediction and sensitivity to therapy in patients with bladder cancer. We scored NRGs in each tissue by using ssGSEA, and selected gene sets that were significantly associated with NRGs scores by using the WCGNA algorithm. Based on the expression profiles of NRGs-related genes, NMF clustering analysis was performed to identify different BLCA molecular subtypes. For the differentially expressed genes between subtypes, we used univariate COX regression, LASSO regression and multivariate COX regression to further construct a hierarchical model of BLCA patients containing 10 genes. This model and the nomogram based on this model can accurately predict the prognosis of BLCA patients in multiple datasets. Besides, BLCA patients classified based on this model differ greatly in their sensitivity to immunotherapy and targeted therapies, which providing a reference for individualized treatment of patients with bladder cancer.

## Introduction

Bladder cancer (BLCA) is one of the top ten most common cancers in the world. In 2022, an estimated 164,190 cases of urological cancer will be diagnosed in the United States, and about 31,990 patients will die from urological cancer, with BLCA accounting for about 50% of these cases, and significantly more men than women^1^. China has an estimated 91,893 BLCA diagnoses and about 42,973 deaths from BLCA in 2022^2^. The number of patients with BLCA continues to increase worldwide, posing a considerable public health burden^3^. Plans to reduce the number of BLCA deaths by reducing the number of patients diagnosed with advanced disease are inadequate. There is an urgent need to explore accurate and reliable novel biomarkers to predict the prognosis of patients with BLCA so as to achieve individualized management and treatment.

Neutrophils play an important role in the occurrence and development of cancer, and their functions are mainly through degranulation, phagocytosis, and the release of neutrophil extracellular traps (NETs)^4^. NETs are reticulated DNA structures decorated with various protein substances (e.g., histones, myeloperoxidase, neutrophil elastase, lactoferrin, etc.) released extracellularly by neutrophils stimulated by strong signals (e.g., various microorganisms, cytokines, proteases, damage-associated molecular patterns, etc.)^5^. Back in 2004, Brinkmann et al.^6^ first reported that neutrophils kill bacteria by releasing NETs. However, in addition to their role in host defense against microbial infection, NETs are equally important as pathological mediators in sterile inflammatory conditions such as cancer, autoimmunity, and ischemia–reperfusion injury (IRI)^5,7^. In the tumor microenvironment (TME), NETs are involved in tumor growth, metastasis, and treatment resistance^8^. Crosstalk between NET formation and TME suggests that NETs contribute to cancer progression and metastasis^9,10^. Study has shown that NET-DNA activates ILK-β-parvin pathway by binding to CCDC25, a transmembrane receptor on cancer cells, to enhance cell motility and thus promote cancer metastasis^11^. In addition, NETs can also inhibit T cell responses through metabolism and functional failure, thereby promoting tumor growth^12^. Studies on the pro-tumor effects of NETs are gradually increasing, and some studies have reported the association of NET-related genes with head and neck squamous cell carcinomas^13^, non-small-cell lung cancer^14^ and renal IRI^15^, however, few studies have focused on the function of NETs in BLCA. Therefore, it is very meaningful to explore NET-related biomarkers to predict the prognosis of BLCA patients and the efficacy of their immunotherapy.

In this study, we comprehensively analyzed the relationship between NET-related genes (NRGs) and BLCA. Each sample was first scored for NETs based on the expression of NRGs in each bladder cancer sample in the TCGA database, and modular genes significantly associated with NETs scores were screened using the WGCNA algorithm. Based on the expression levels of these genes, we used the NMF machine learning approach to classify BLCA samples into two subgroups with different clinical features, tumor microenvironment and immune characteristics. Subsequently, based on differentially expressed genes (DEGs) between subgroups, we further constructed a prognostic risk model containing 10 genes, and the nomogram constructed based on this model can accurately predict the prognosis of BLCA patients in the TCGA database. In addition, bladder cancer patients classified according to this signature differed significantly in their sensitivity to immunotherapy and targeted therapy. Finally, we further determined that the CPT1C gene was closely associated with the development of BLCA.

## Materials and methods

### Data collection and processing

Firstly, we downloaded the RNA sequencing data, somatic mutation data, gene copy data and corresponding clinical information of BLCA patients from The Cancer Genome Atlas (TCGA) database. Similarly, the gene expression matrix and clinical information of BLCA patients in GSE13507 and GSE32894 microarray datasets were obtained from Gene Expression Omnibus (GEO) database. Using IMvigor210CoreBiology R package (http://research-pub.gene.com/IMvigor210CoreBiologies) to obtain transcriptome data and clinical information of patients with metastatic urothelial carcinoma who received immunotherapy to validate the performance of the model we constructed to predict the efficacy of immunotherapy^16^. A total of 231 neutrophil extracellular trap-related genes (NRGs) analyzed in this study were summarized from previous studies. Samples without complete transcriptome data or clinical information in each dataset were excluded from further analysis. The RNA sequencing data in the TCGA-BLCA dataset were transformed into log2(TPM + 1) to maintain comparability with the microarray dataset. The “sva” R package was used to eliminate the batch effect between microarray datasets^17^.

### Construction of neutrophil extracellular traps (NETs) scores and co-expression network

Based on the expression of 231 NRGs in the TCGA, GSE13507, and GSE32894 datasets, the "GSVA" R package was used to calculate NETs scores for each sample. Weighted Gene co-expression network analysis (WGCNA) was used to identify gene modules with similar expression patterns and calculate the correlation between gene modules and NETs scores^18^. A hierarchical clustering tree was established by dynamic hybrid cutting method to identify co-expressed gene modules and each leaf of the tree represented a gene. Genes with similar expression pattern are clustered to form a branch of the tree, and each branch represents a gene module. Using Pearson correlation analysis to calculate the correlation between gene module and NETs scores and intersecting the genes in the modules most relevant to the NETs scores in the three datasets.

### Non-negative matrix factorization (NMF) algorithm

According to the intersection genes obtained by WGCNA analysis, the NMF algorithm was used to cluster the BLCA samples in TCGA dataset. The “brunet” criterion was selected and iterated 100 times. We set the number of clusters (k) from 2 to 10 and set the minimum number of members of each cluster to 25. The R package “NMF” was used to determine the average contour width of the common membership matrix^19^. The cophenetic correlation coefficients (from 0 to 1) were used to reflect the stability of clusters, while the residual sum of squares (RSS) was used to reflect the model’s clustering performance. The optimal k was selected based on the cophenetic, dispersion, and silhouette metrics. BLCA samples were eventually divided into different molecular clusters through the above algorithm and the optimal k.

### Evaluation of tumor microenvironment and immune invasion

Using "ESTIMATE" R package to estimate the tumor microenvironment, and the strimal score, immune score, ESTIMATE score and tumor purity of each sample were obtained^20^. Based on the marker genes of 23 immune cells from the TISIDB database (http://cis.hku.hk/TISIDB/), the "GSVA" R package was used to evaluate the infiltration of 23 immune cells in each sample.

### Functional enrichment analysis

Gene Ontology (GO) and Kyoto Encyclopedia of Genes and Genomes (KEGG) pathway enrichment analysis was performed through the "clusterProfiler" R package^21^. GSEA analysis was used to compare significantly different biological processes between different subgroups of BLCA patients. Pathways with FDR < 0.25 and *p* < 0.05 are considered statistically enriched^22^. Using the "GSVA" R package for ssGSEA analysis to evaluate the activity scores of specific biological pathways in each sample. Spearman correlation test was performed to calculate the correlation between risk score and pathway activity score, and screen out the pathways with a correlation coefficient > 0.3. The reference gene set included in a specific biological pathway was obtained from the MSiDB database (https://www.gsea-msigdb.org/gsea/msigdb/index.jsp).

### Prediction of the immunotherapy response and drug sensitivity

The Tumor Immune Dysfunction and Rejection (TIDE) score was used to predict the immune escape potential and immunotherapy efficacy of each BLCA patient. The higher the TIDE score, the greater the tumor's immune escape potential, and less likely the patient can benefit from immunotherapy^23^. The immune appearance score (IPS) of each BLCA sample was obtained from the Cancer Immunology Atlas (TCIA) database to predict the patient's response to immunotherapy with PD-1 and CTLA4 blockers^24^. Besides, we also validated the association between patient risk characteristics and immunotherapy benefits using the IMvigor210 immunotherapy dataset.

The "pRRophic" R package was used to predict the sensitivity of BLCA patients to targeted therapy drugs. Specifically, the Gene expression profiling and IC50 value of cancer cells under the treatment of corresponding drug in the GDSC database were used as reference, and the IC50 values of targeted drugs were estimated according to the Gene expression profiling of BLCA samples through tenfold cross-validated ridge regression.

### Calculation of tumor mutation burden (TMB) and copy number variation (CNV)

The TMB represents the number of mutations per million bases in tumor tissue, including genetic coding errors, base substitutions, base insertions, and deletions. In theory, tumor tissues with higher TMB are easier to be recognized by the immune system, so immunotherapy against them may be more effective. Therefore, we calculated the TMB score of each BLCA patient according to the somatic cell mutation data obtained from the TCGA database. Besides, based on the CNV data downloaded from the TCGA database, we also calculated the CNV frequency of the corresponding genes and displayed the results in the form of a lollipop plot.

### Construction of the neutrophil extracellular trap-related prognostic stratification model for BLCA patients

Differential expression analysis was performed on the two different BLCA clusters obtained by NMF algorithm, |log2FC|> 1 and FDR < 0.05 set as threshold. Subsequently, the TCGA dataset was randomly divided into a training set and a validation set at a ratio of 7:3. In the training set, univariate cox regression analysis was performed to identify genes with good prognostic ability (HR ≠ 1 and *p* value < 0.05). To further reduce the number of candidate genes, the LASSO regression algorithm in the “glmnet” R package was used to eliminate overfitting bias through tenfold cross-validation to obtain a more concise prognostic gene combination^25^. Finally, multivariate Cox regression analysis was used to construct the final prognostic model. Exporting the coefficient value for each gene in the multivariate Cox regression, and the risk score is equal to the expression level of each gene multiplied by its corresponding regression coefficient.

$$Risk\;score = \sum\limits_{i = 1}^{n} {\left( {{\text{coef}}_{i} \times {\text{Exp}}_{i} } \right)}$$

The time-dependent receiver operating characteristic curve (ROC) was used to evaluate the predictive ability of the above risk model. The K–M survival curve was used to compare the survival differences of BLCA patients in different risk groups. Then, the accuracy of the risk model was verified using the same method in the GSE13507 and GSE32894 datasets.

### Independent prognostic analysis and construction of nomogram

To evaluate whether risk score is an independent prognostic factor for BLCA patients, we conducted univariate and multivariate cox regression analysis on other clinical information and risk score of BLCA patients to screen for independent prognostic factors. Using the "regplot" R package to integrate the above independent prognostic factors to construct a clinically applicable prognostic nomogram, and the calibration curve and decision curve analysis (DCA) were used to evaluate the accuracy of the nomogram.

### Statistical analysis

All statistical analyses involved in this study were performed by R software (version 4.1.0). Continuous variables were compared using the student's t-test, and non-normally distributed variables were compared using the Wilcoxon rank sum test. Chi-square test was used to compare the two groups of Categorical variables. All statistical tests were two-sided, and *p* value < 0.05 was considered statistically significant.

## Results

### Identification and enrichment analysis of gene modules related to NETs score in BLCA

**Figure 1:**
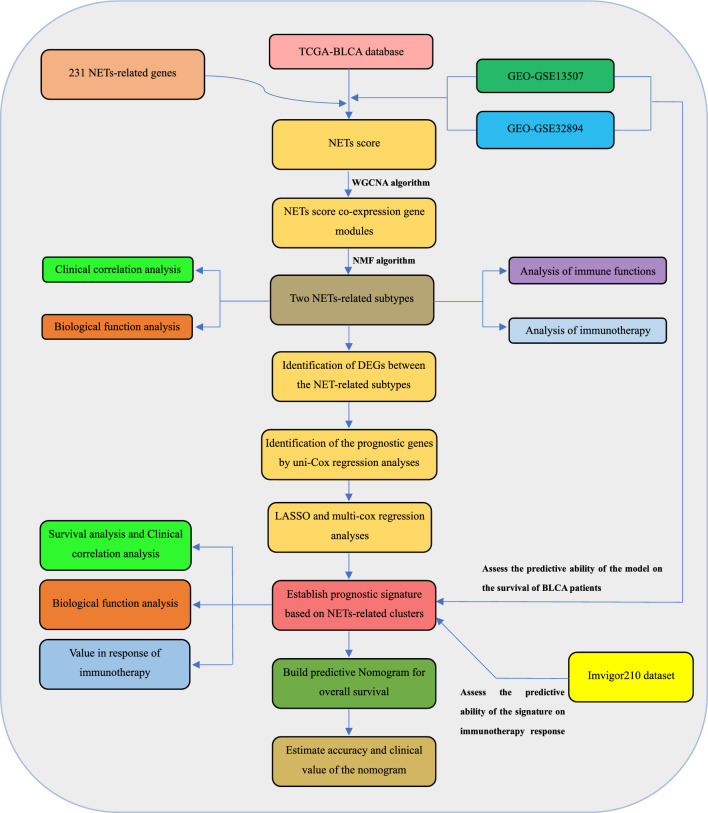
The flowchart of this study.

**Figure 2:**
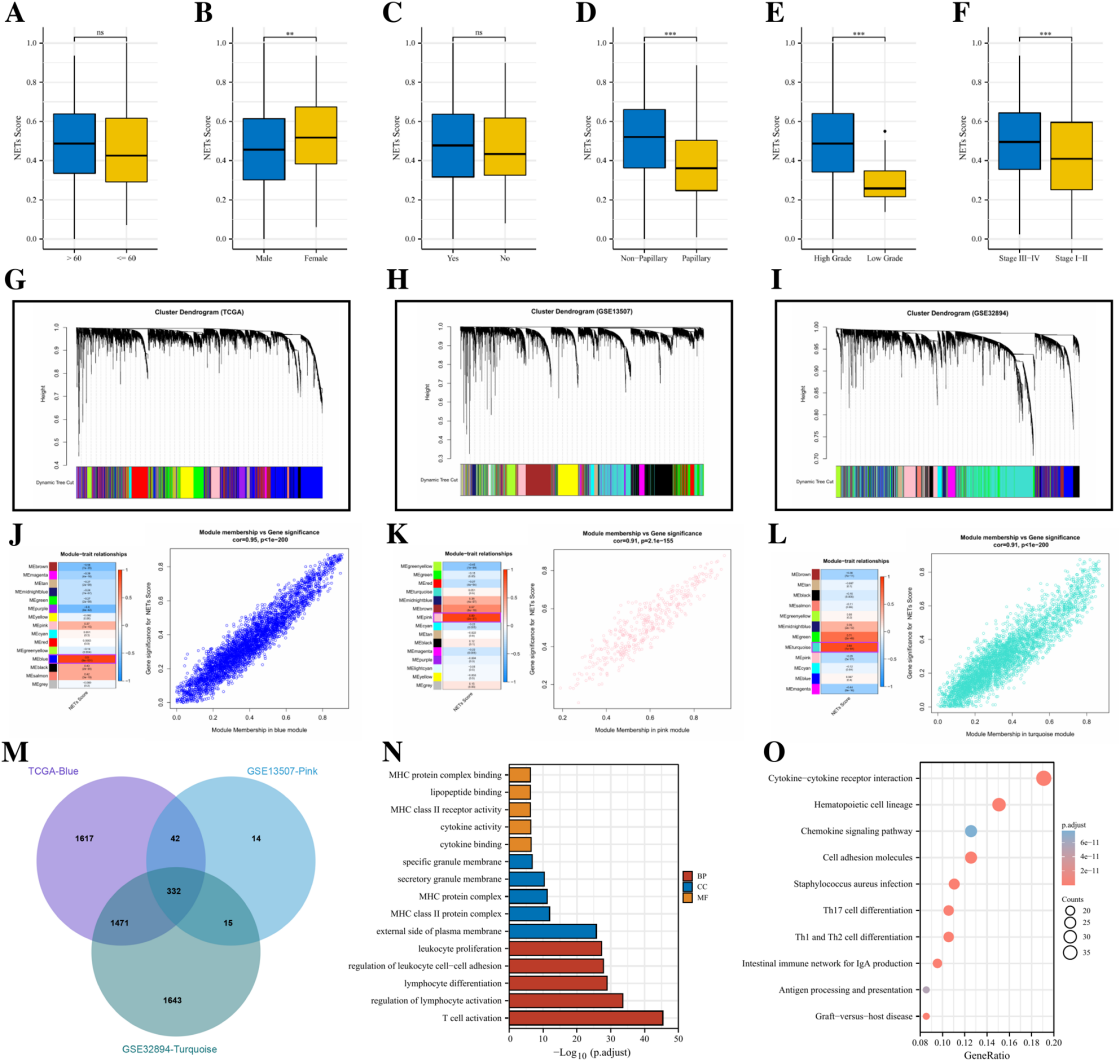
The comparisons of NETs-score between different subgroups. age (**A**), gender (**B**), smoking status (**C**), cancer subtype (**D**), histologic grade (**E**), clinical stage (**F**). The co-expression network was established using weighted gene co-expression network analysis methods based on the RNA-seq profiles from (**G**) the TCGA-BLCA database, (**H**) GSE13507 dataset, and (**I**) GSE32894 dataset. Heatmap demonstrating the correlation between module eigengenes and NETs score in the (**J**) TCGA-BLCA dataset, (**K**) GSE13507 dataset, and (**L**) GSE32894 dataset. (**J**) The blued module had the strongest correlation with NETs score in the TCGA-BLCA dataset (Cor = 0.95, *p* < 1e^−200^). (**K**) The pink module had the strongest correlation with NETs score in the GSE13507 cohort (Cor = 0.91, *p* = 2.1e^−155^). (**L**) The turquoise module had the strongest correlation with NETs score in the GSE32894 dataset (Cor = 0.91, *p* < 1e^−200^). (**M**) Venn diagram displaying the NETs score-related selected intersection genes from different datasets. (**N**, **O**) Gene ontology (GO) and Kyoto Encyclopedia of Genes and Genomes (KEGG) analyses of NETs score-related intersecting genes. *ns* not significant; *p < 0.05; **p < 0.01; ***p < 0.001.

Figure 1 shows the flow chart for this study. NRGs for ssGSEA analysis are listed in Supplementary Table S1. Figure 2A–F shows the relationship between NETs score and patient age, gender, smoking status, subtype, pathological grade, and clinical stage in the TCGA-BLCA cohort. It can be seen that the NETs score of female patients is significantly higher than that of male patients. Besides, the higher the malignancy (No-palillary, High grade, and Late stage), the higher the NETs score seems to be. This indicates that NETs score may be associated with the malignant progression of bladder cancer.

To further screen for genes significantly correlated with NETs scores, we conducted WCGNA analysis. In the TCGA cohort (Fig. 2G, J), the blue module was highly correlated with NETs scores among 15 modules (R^2^ = 0.95, *p* < 1e^−200^). In the GSE13507 cohort (Fig. 2H, K), the pink module was highly correlated with NETs scores among the 15 modules (R^2^ = 0.91, *p* = 2.1e^−155^). In the GSE32894 cohort (Fig. 2I, L), turquoise module was highly correlated with NETs scores among the 12 modules (R^2^ = 0.91, *p* < 1e^−200^). A total of 332 NETs score-related genes were identified by intersecting the above 3 core gene modules (Fig. 2M). The results of GO enrichment analysis showed that these NETs score-related genes were mainly enriched in immune-related biological processes, such as T cell activation, regulation of lymphocyte activation, lymphocyte differentiation, etc. (Fig. 2N). Besides, the results of KEGG pathway enrichment analysis were also enriched in immune-related pathways such as Cytokine-cytokine receptor interaction, Chemokine signaling pathway, Th17 cell differentiation, Th1 and Th2 cell differentiation, and Antigen processing and presentation (Fig. 2O).

### Clinical characteristics of NETs-related clusters

**Figure 3:**
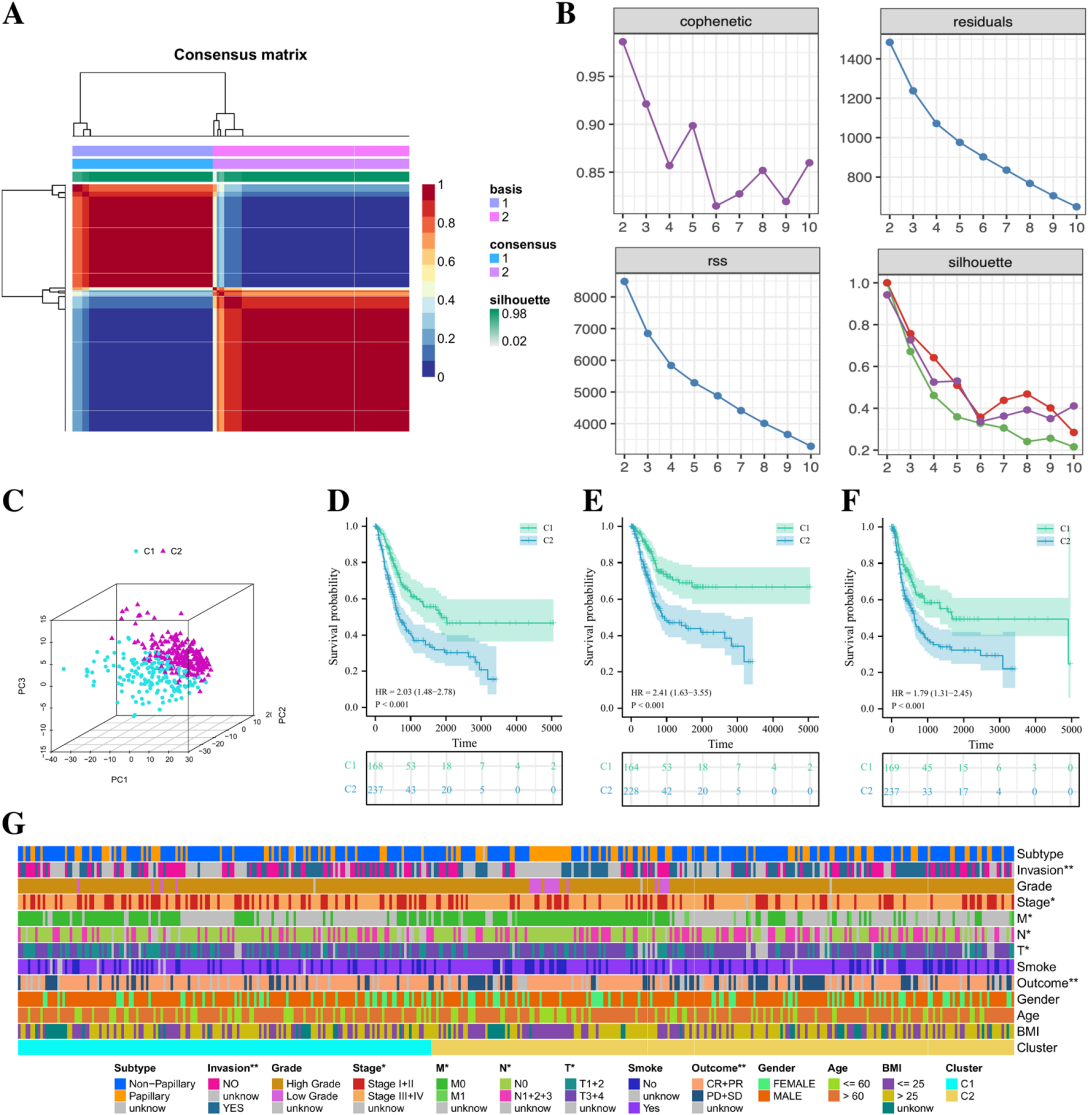
Cluster analysis of intersection genes related to NETs-score in the TCGA cohort. (**A**, **B**) According to the steepness of the “cophenetic” decline, the BLCA dataset in the TCGA cohort was divided into two distinct clusters. (**C**) The 3D PCA plots showed the clusters could distinguish BLCA patients based on the expression profiles of NETs-score related genes. (**D**, **E**, **F**) The Kaplan–Meier curve survival analysis of Overall Survival (OS), Disease-Specific Survival (DSS) and Progress-Free Interval (PFI) between different cluster groups. (**G**) The clinical pathological characteristic heatmap between different cluster groups. *p < 0.05; **p < 0.01.

Based on the above NETs-score related genes, we used the NMF algorithm to cluster TCGA-BLCA patients and based on the steepness of the “cophenetic” decline, the optimal number of clusters selected was 2 (Fig. 3A, B). The results of PCA analysis showed that 169 C1 patients classified based on NETs-score related genes were significantly different from 237 C2 patients in Gene expression profiling (Fig. 3C). Prognostic analysis of the two subtypes of BLCA patients showed that the OS (HR = 2.03, *p* < 0.001), DSS (HR = 2.41, *p* < 0.001) and PFI (HR = 1.79, *p* < 0.001 ) of the C2 group were significantly worse than the C1 group (Fig. 3D–F). The clinical pathological characteristic heatmap showed that some clinical features of patients in C1 and C2 groups were also different, including tumor invasion, clinical stage, tumor M stage, T stage, N stage, and treatment benefits (Fig. 3G).

### Biological characteristics of NETs-related clusters

**Figure 4:**
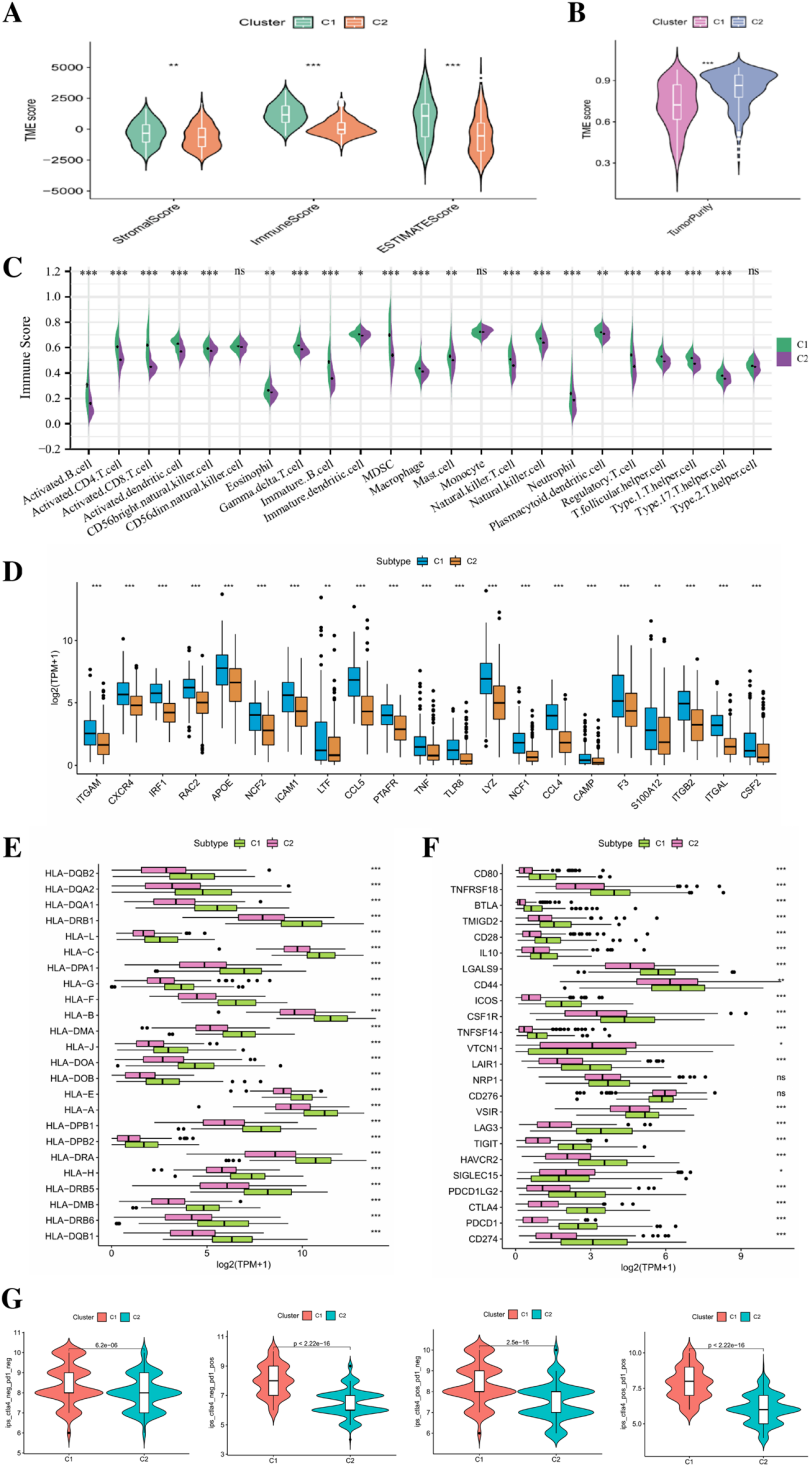
The comparisons of the stromal score, immune score, and ESTIMATE score between different clusters. (**B**) The comparisons of tumor purity between different clusters. (**C**) The pod plot displaying the difference in immune cell infiltration between different clusters. (**D**) The boxplot illustrating the difference in the expression of differentially expressed NRGs between different clusters. (**E**) The boxplot illustrating the expression of HLA-related genes between different clusters. (**F**) The boxplot illustrating the expression of immune checkpoint genes between different clusters. (**G**) The comparison of immunophenotype score (IPS) between different cluster groups including CTLA4^−^_PD1^−^, CTLA4^−^_PD1^+^, CTLA4^+^_PD1^−^, CTLA4^+^_PD1^+^. *ns* not significant; *p < 0.05; **p < 0.01; ***p < 0.001.

To verify the effectiveness of the above clustering method, we compared the tumor microenvironment and immune cell infiltration of the two groups. As for tumor microenvironment, the stromal score, immune score and ESTIMATE score of C2 group were significantly lower than that of C1 group, while the tumor purity of C2 group was significantly higher than that of C1 group (Fig. 4A, B). The results of immune cell infiltration analysis confirmed the above results. A variety of immune cells including B cells, CD4^+^T cells, CD8^+^T cells, regulatory T cells, macrophages were significantly more abundant in the C1 group (Fig. 4C). Besides, we also compared the expression levels of NRGs, HLA-related genes and immune checkpoint-related genes (ICGs) in the two BLCA subgroups. Figure 4D shows that the differentially expressed NRGs genes are mainly highly expressed in the C1 group. Figure 4E shows that a variety of HLA-related genes are mainly highly expressed in the C1 group, whereas a variety of ICGs are mainly highly expressed in C2 group (Fig. 4F). IPS analysis can be used to predict the ability of tumors to respond to ICIs. As shown in Fig. 4G, the four IPS scores of C1 group are all higher than that of C2 group, which indicates that C1 group have stronger tumor immunogenicity and higher immune activity in tumor microenvironment. Correspondingly, immunotherapy may be more beneficial for the patient belonging to C1 subtype.

**Figure 5:**
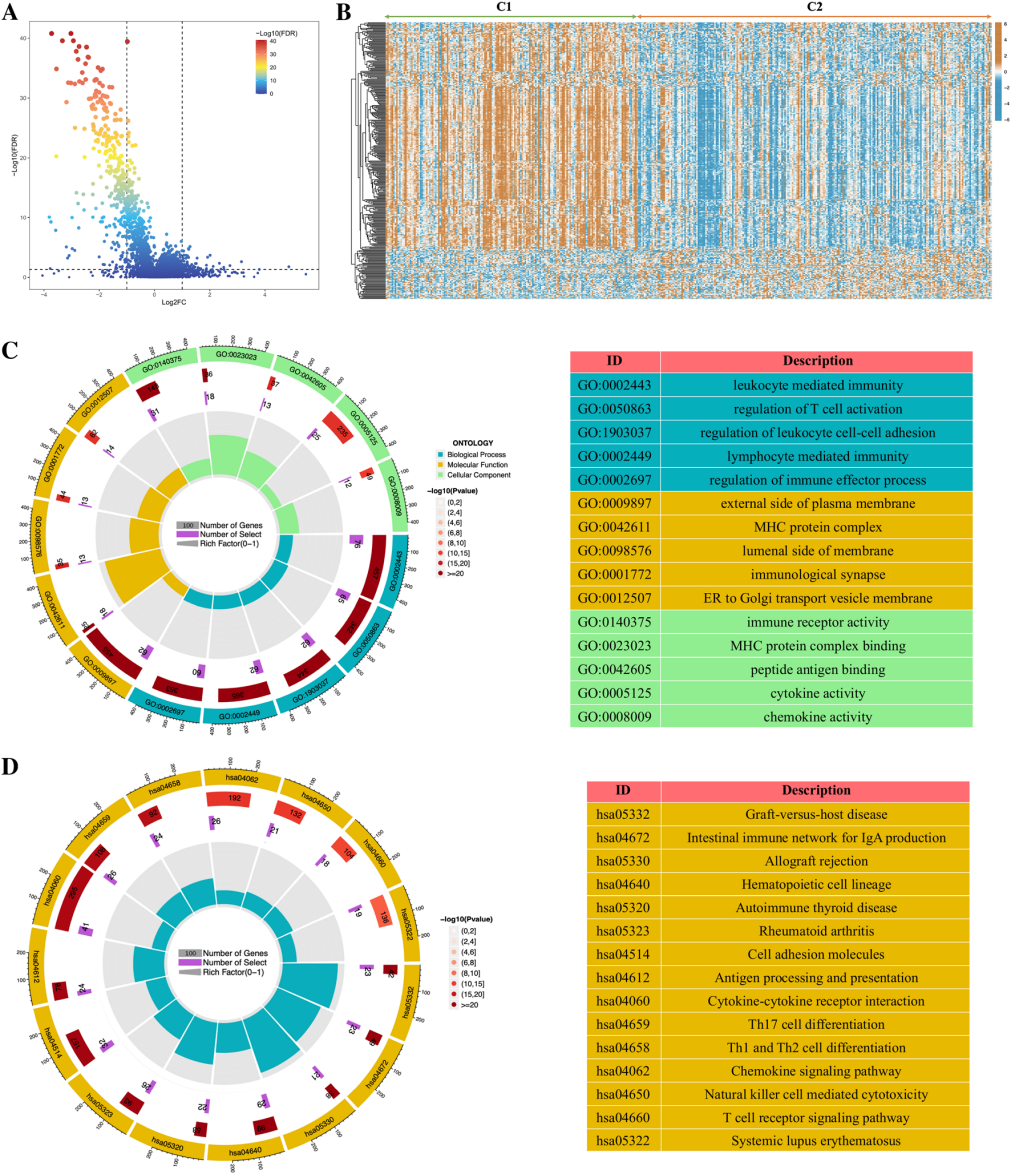
The volcano plot (**A**) and the heatmap (**B**) showing the differentially expressed genes (DEGs) between different clusters. (**C**) The GO analysis of the DEGs between different clusters. (**D**) The KEGG pathway enrichment analysis of DEGs between different clusters.

In order to further explore the underlying mechanism of these differences, we conducted a differential analysis of the Gene expression profiling of the two subgroups and finally obtained 358 differentially expressed genes (DEGs). The volcano plot of the differential analysis and the heatmap of 358 DEGs between the two groups are shown in Fig. 5A, B. Subsequently, we conducted GO and KEGG enrichment analysis on these 358 DEGs. The results of GO enrichment analysis mainly focus on immune related biological processes such as leukocyte mediated immunity, regulation of T cell activation, and regulation of immune effects (Fig. 5C). The results of KEGG pathway enrichment analysis also include many immune regulation-related pathways, such as Cytokines-cytokine receptor interaction, intestinal immune network for IgA production, Th17 cell differentiation, antigen processing and presentation, and T cell receptor signaling pathway (Fig. 5D). The above results indicate that there are significant differences in tumor immunity between the two subgroups classified by the NMF algorithm.

### Construction and validation of NETs-related risk model

**Figure 6:**
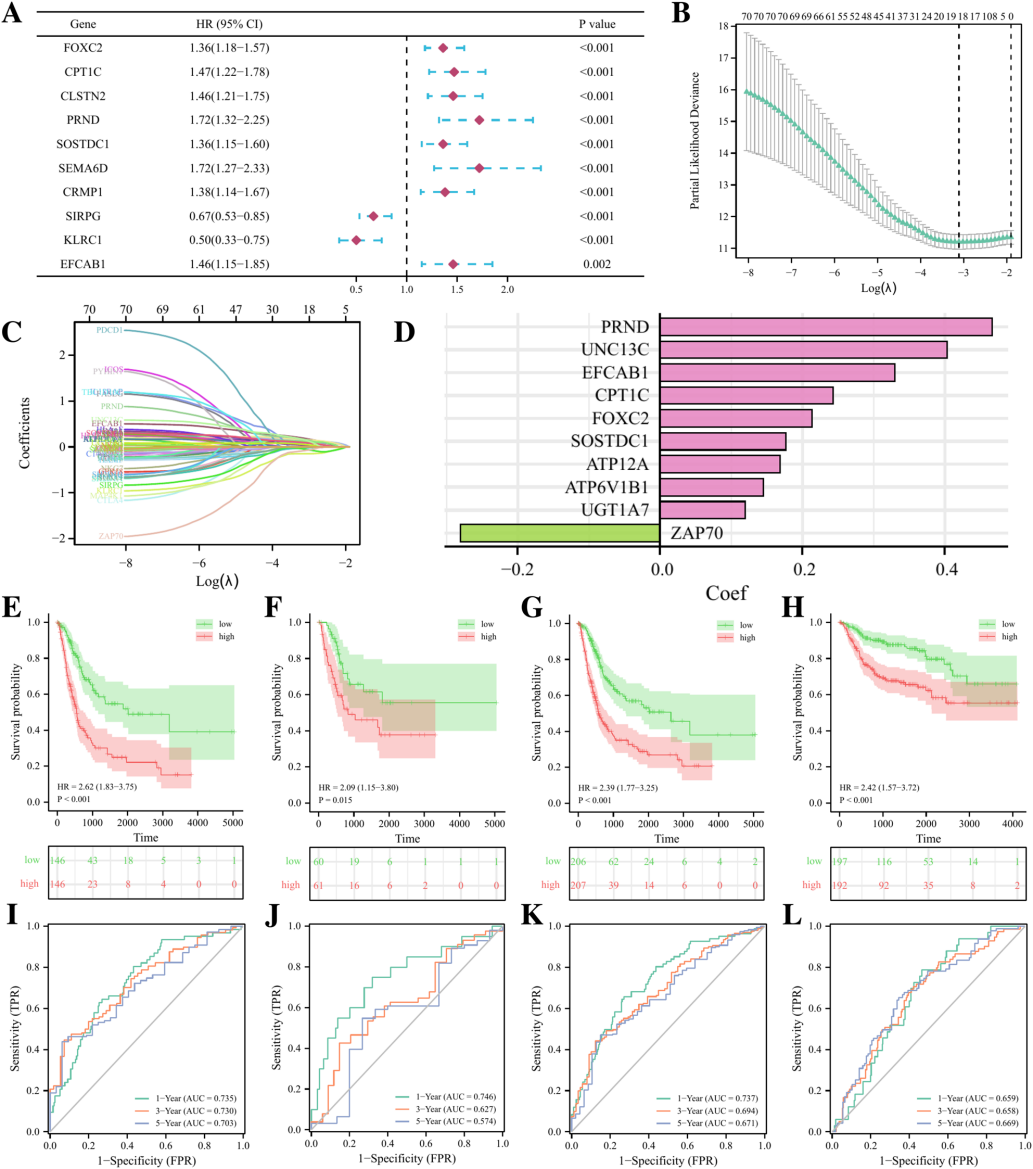
Development and validation of the NETs-related signature. (**A**) The forest plot displaying the HR (95% CI) and *p* values of prognostic genes (top 10, according to *p* value). (**B**, **C**) 19 candidate genes were obtained by LASSO regression analysis. (**D**) The coefficients of the finally 10 genes in the signature. The Kaplan–Meier survival curves for BLCA patients stratified by the risk score in the TCGA training set (**E**), the TCGA validation set (**F**), the overall TCGA set (**G**), the external validation set (GSE3507 and GSE32894 merged) (**H**). ROC analysis at 1, 3, and 5 years of BLCA patients in the TCGA training set (**I**), the TCGA validation set (**J**), the overall TCGA set (**K**), and the external validation set (GSE3507 and GSE32894 merged) (**L**).

To better apply the above subgroup classification for clinical treatment guidance and to quantify the specific prognostic risk for each BLCA patient. We aim to construct a hierarchical model based on the above differentially expressed genes to help the prognosis prediction and treatment decision-making of BLCA patients. Firstly, we performed univariate cox regression on the above DEGs in the training set, and identified 71 genes with significant prognostic value. Figure 6A shows the top ten prognostic genes (sorted by p-value). Then, we used LASSO regression analysis to sub-screen the 71 genes mentioned above to streamline the final risk model (Fig. 6B, C) and determined the optimal λ value with the minimum probability deviation through ten-fold cross-validation. According to the optimal λ value, we obtained 19 candidate genes (Fig. 6B, C). Multivariate cox regression analysis was performed on these genes to construct the final model. The risk score (RS) of each BLCA sample was the expression level of ten genes included in the final model multiplied by the coefficient of their multivariate cox regression (Fig. 6D).

According to the median value of RS in the training set, we divided BLCA patients in the TCGA training set, TCGA validation set, and external validation set (combined set including GSE3507 and GSE32894) into high and low risk groups. The results of K-M survival analysis showed that the OS of patients in the high-risk group was significantly worse than that in the low-risk group no matter in which cohort (Fig. 6E–H). In the TCGA training set, the AUC of the OS at 1 year, 3 year, and 5 year was 0.735, 0.730, and 0.703, respectively. In the TCGA validation set, the AUC of the OS at 1 year, 3 year, and 5 year was 0.746, 0.627, and 0.574, respectively. As for the whole TCGA set, the AUC of the OS at 1 year, 3 year, and 5 year was 0.737, 0.694, and 0.671. For the external validation set, the AUC of the OS at 1 year, 3 year, and 5 year was 0.659, 0.658, and 0.669 (Fig. 6I–L). The above results all indicate that our risk model has a strong predictive effect.

### Correlation analysis of clinical characteristics and construction of nomogram

**Figure 7:**
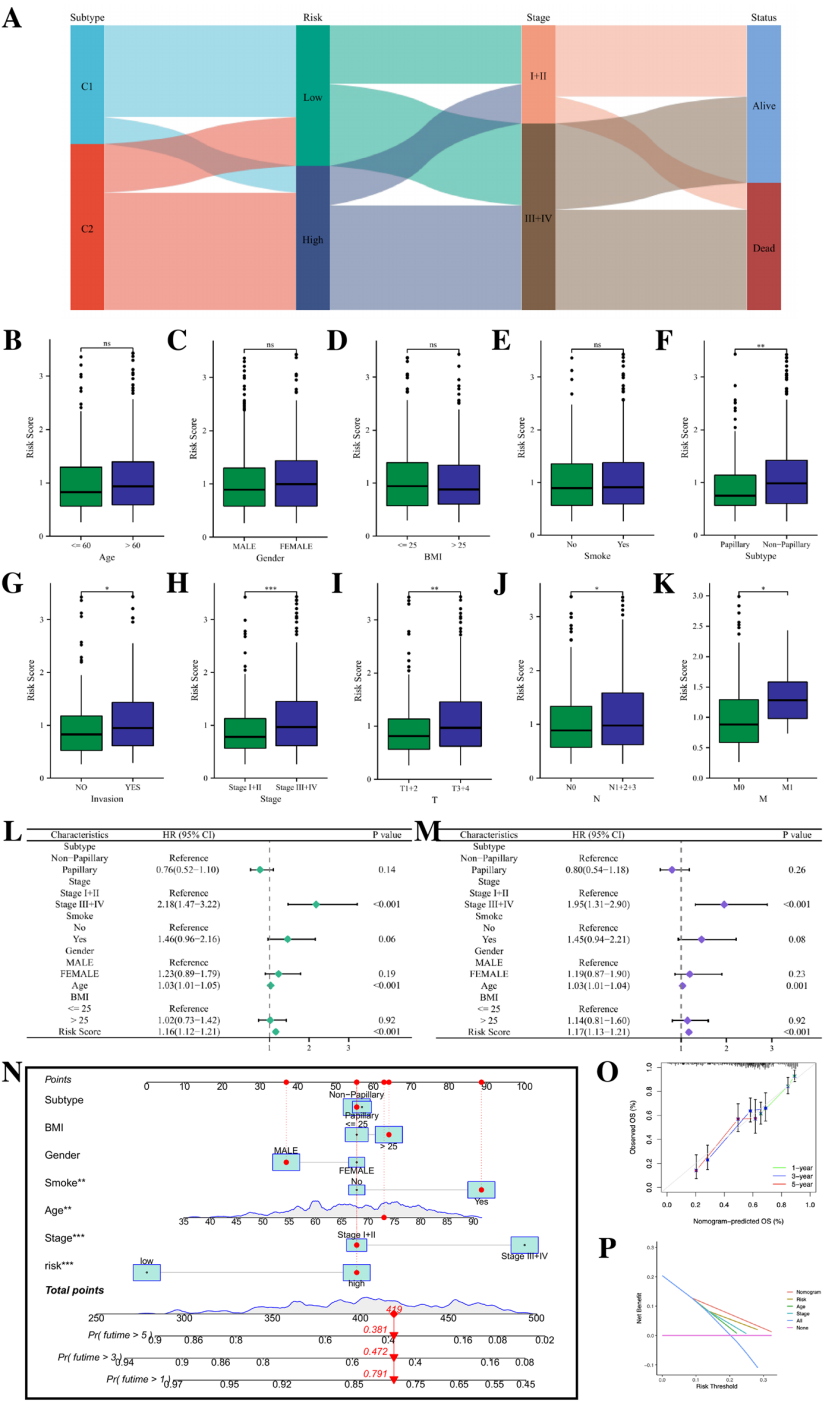
(**A**) The Sankey diagram revealed the potential connection between NMF cluster, risk score, clinical stage, and survival status. (**B**–**K**) The comparisons of the risk score in BLCA patients with different age, gender, BMI, smoking status, cancer subtypes, tumor invasion, clinical stage, T stage, N stage and M stage. (**L**, **M**) Univariate and multivariate Cox regression analyses showed that risk score is an independent prognostic factor of BLCA. (**N**) The nomogram combining risk score and other clinicopathological parameters was developed to predict 1-, 3-, and 5-year survival. (**O**) Calibration curves showing the predictions of the nomogram that we established for 1-, 3-, and 5-year overall survival. (**P**) The DCA curves showing the predictions of the nomogram, risk score and other clinicopathological parameters. *ns* not significant; *p < 0.05; ***p < 0.001.

The Sankey diagram intuitively reflected the changes of BLCA patients in different classification modes (Fig. 7A). The results of clinical correlation analysis showed that patients with non-papillary tumors, tumor with lymphatic vascular invasion, late clinical stage, and higher TNM stage had higher risk scores, while other clinical characteristics such as age, gender, BMI, and smoking did not seem to be related to risk scores (Fig. 7B–K). To determine whether the risk score is an independent prognostic factor for BLCA patients, we performed univariate and multivariate cox analysis, and the results confirmed that the risk model based on 10 gene expression is an independent prognostic factor for BLCA patients. Besides, patient age and tumor clinical stage were also found to be independent prognostic factors for BLCA patients (Fig. 7L, M). To provide a more accurate clinical prediction scheme, we constructed a prognostic nomogram by integrating multiple clinical information and our risk model, which can intuitively display the estimated survival at 1-year, 3-year and 5-year of BLCA patients (Fig. 7N). It can be seen from the calibration plot that there is a good consistency between the patient survival predicted by the nomogram and the actual survival (Fig. 7O). The decision curve analysis (DCA) shows that the nomogram we constructed has the highest accuracy in predicting the prognosis of BLCA patients (Fig. 7P).

### Enrichment analysis based on risk model

**Figure 8:**
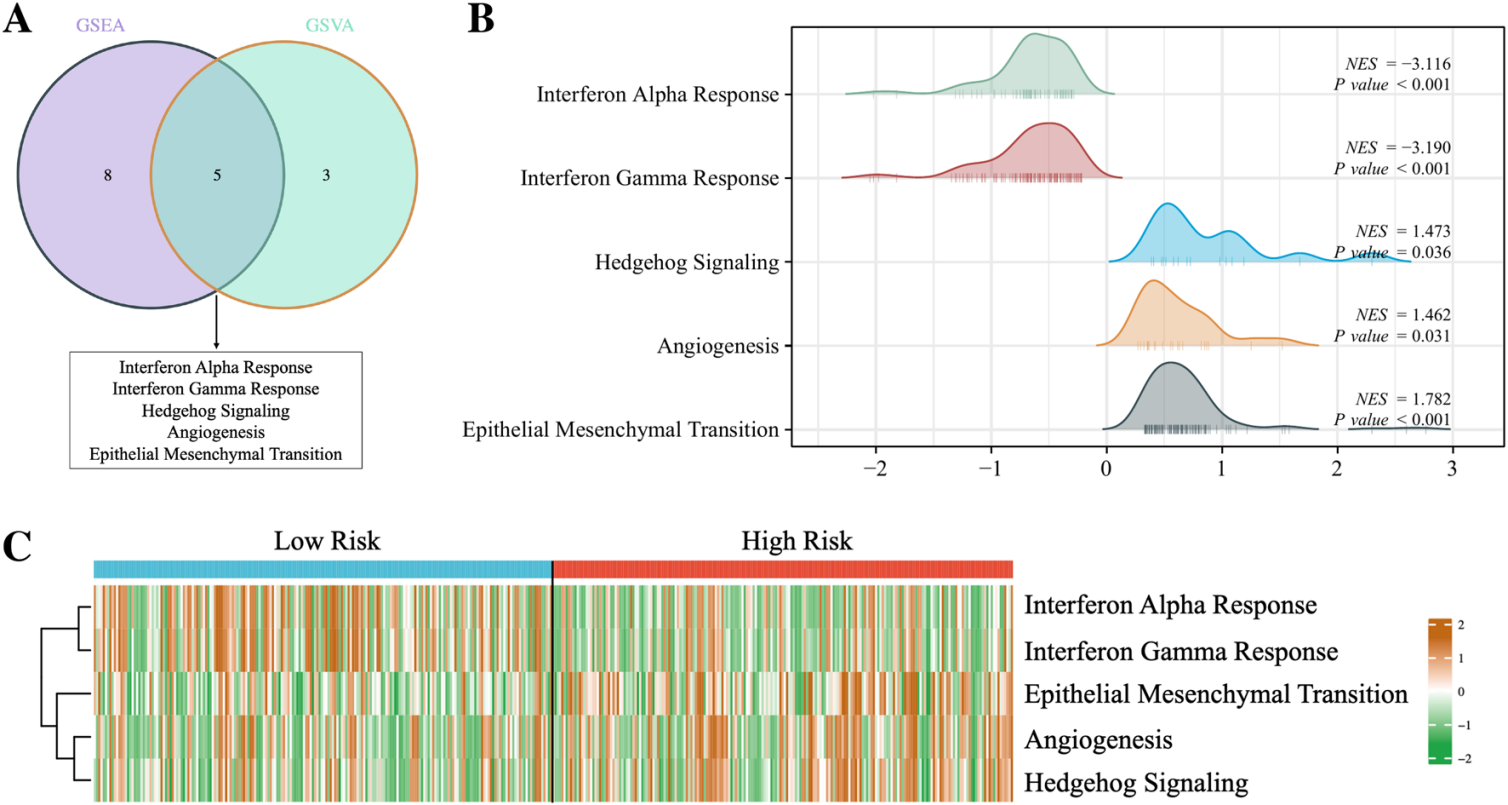
Venn diagram displaying a total of five pathways resulting from the intersection of GSVA enrichment analysis with GSEA enrichment analysis based on differentially expressed genes in different risk groups. (**B**) GSEA enrichment analysis of the five pathways. (**C**) The heat map displaying GSVA enrichment scores of the five pathways of each patient.

To further explore the potential biological mechanisms that lead to many differences between the high-risk group and the low-risk group, we performed GSEA enrichment analysis and GSVA enrichment analysis simultaneously based on the Hallmarks gene set (h.all.v7.2.symbols.gmt) in the MSigDB database. The results of two enrichment analysis showed that compared with the low-risk patients, the high-risk patients had obvious activation of hedgehog signaling pathway, angiogenesis and EMT, while the low-risk patients had the characteristics of activation of INF-a and INF-b response signaling pathways (Fig. 8A–C). The results of the GSEA analysis are shown in Fig. 8B, and the heatmap of the GSVA enrichment of the five characteristics is shown in Fig. 8C.

### Guiding significance of risk model for immunotherapy of BLCA patients

**Figure 9:**
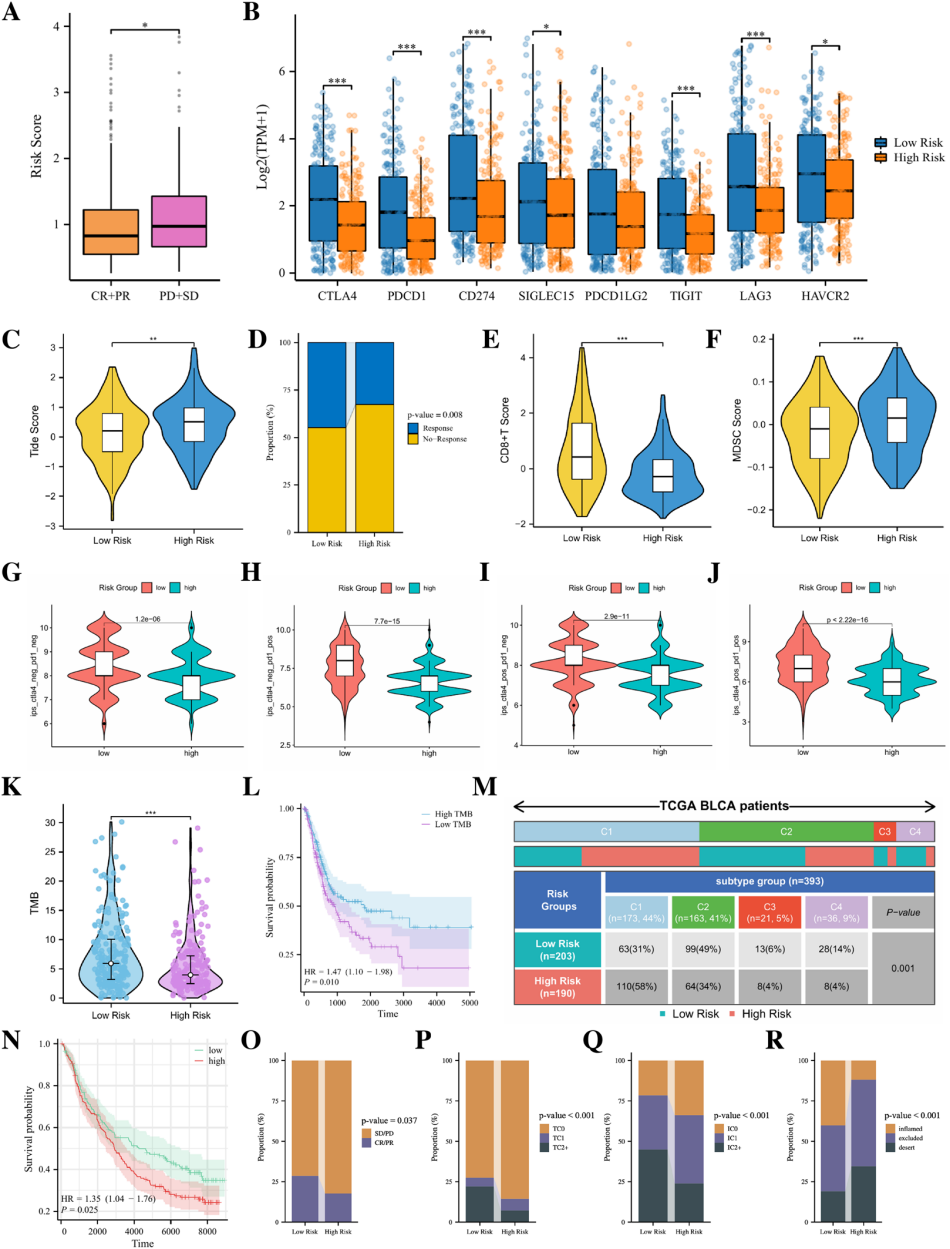
(**A**) The comparisons of the risk score in TCGA-BLCA patients with treatment outcomes. (**B**) The boxplot displaying the difference in common immune checkpoint genes between different risk groups. The comparisons of the TIDE score (**C**), immunotherapy response proportion (**D**), CD8^+^ T score, (**E**) MDSC score (**F**) between different risk groups. The comparison of immunophenotype score (IPS) between different risk groups. (**G**) CTLA4^−^_PD1^−^, (**H**) CTLA4^−^_PD1^+^, (**I**) CTLA4^+^_PD1^−^, (**J**) CTLA4^+^_PD1^+^. (**K**) The comparisons of tumor mutational burden (TMB) of patients in different risk groups. (**L**) The Kaplan–Meier survival curve between the high- and low-TMB groups. (**M**) Comparison of the differences in immune subtype between different risk groups. (**N**) The Kaplan–Meier survival curve between the high- and low-risk groups in the IMvigor210 cohort. Predictive value of risk score for immunotherapy response in the IMvigor210 cohort. (**O**) The percentage of immunotherapy response among risk groups of patients in the IMvigor210 cohort. (**P**) The percentage of tumor cell (TC) level type among risk groups of patients in the IMvigor210 cohort. (**Q**) The percentage of immune cell (IC) level type among risk groups of patients in the IMvigor210 cohort. (**R**) The percentage of immune subtypes among risk groups of patients in the IMvigor210 cohort. Specimens were scored as immunohistochemistry IC0, IC1, IC2, or IC3 if < 1%, ≥ 1% but < 5%, ≥ 5% but < 10%, or ≥ 10% of IC were PD-L1 positive, respectively. Specimens were scored as immunohistochemistry TC0, TC1, TC2, or TC3 if < 1%, ≥ 1% but < 5%, ≥ 5% but < 50%, or ≥ 50% of TC were PD-L1 positive, respectively. *ns* not significant; *p < 0.05; **p < 0.01; ***p < 0.001.

In the TCGA cohort, 357 patients received one or more treatment measures, including 256 CR/PR patients and 101 PD/SD patients. Comparing the risk scores of the two categories of patients, we found that the risk scores of PD/SD patients were significantly higher than those of CR/PR patients (Fig. 9A). In order to provide better individualized treatment guidance for BLCA patients, we comprehensively analyzed the role of our risk model in immunotherapy. As shown in Fig. 9B, a variety of classical immune checkpoint molecules, including CD274, PDCD1, CTLA4 and SIGLEC15, are more highly expressed in low-risk patients, which seems to suggest that administering corresponding immune checkpoint inhibitors to such patients is more beneficial. Based on the TIDE algorithm, we observed that low-risk patients had lower Tide scores and MDSC cell scores than high-risk patients, while their CD8^+^ T cell scores were higher than high-risk patients, indicating that low-risk patients were less likely to evade immunity and immunotherapy for them may be more effective (Fig. 9C, E, F). For patients received immunotherapy, we can see that the percentage of patients in the low-risk group responding to treatment was higher than that of high-risk group (Fig. 9D). As for the IPS score, we found that all four IPS scores of low-risk group were higher than those of high-risk group, which further indicated that low-risk patients may be more sensitive to immunotherapy (Fig. 9G–J). We also analyzed the relationship between risk score and TMB, which is considered to be another potential indicator for evaluating immunotherapy. It has been reported that the higher the TMB of a tumor tissue, the more neoantigens it exposes, and therefore it is easier for the immune system to recognize and clear it. It can be seen from Fig. 9K that the TMB of low-risk patients is higher than that of high-risk patients, which further confirms that patients in the low-risk group may be more sensitive to immunotherapy. Besides, the results of K-M analysis also showed that the survival of patients with low TMB was also worse than that of patients with high TMB (Fig. 9L). Figure 9M shows the proportion of TCGA immune subtypes in patients with different risk groups, and the results indicate that there are indeed significant differences in immunophenotypes between different groups.

We further validated the response to immunotherapy in the IMvigor210 cohort. Figure 9N shows that the risk score calculated based on the 10-gene model can divide the whole patients in this cohort into high-risk and low-risk groups. And consistent with the TCGA cohort as well as the GEO cohort, the high-risk group in the IMvigor210 cohort had worse OS than the low-risk group. Analysis of the results of immunotherapy showed that patients in the high-risk group were less responsive to immunotherapy than patients in the low-risk group (Fig. 9O). Compared to the low-risk group, the percentage of immune cells and tumor cells expressing PD-L1 in the high-risk group was lower (Fig. 9P, Q). The results of inflammatory immune subtype analysis showed that the high-risk group had higher percentages of “immune excluded” and “immune desert” tumors and a lower percentage of "immune inflamed" tumors (Fig. 9R).

### Guiding significance of risk model for chemotherapeutic and targeted therapy of BLCA patients

**Figure 10:**
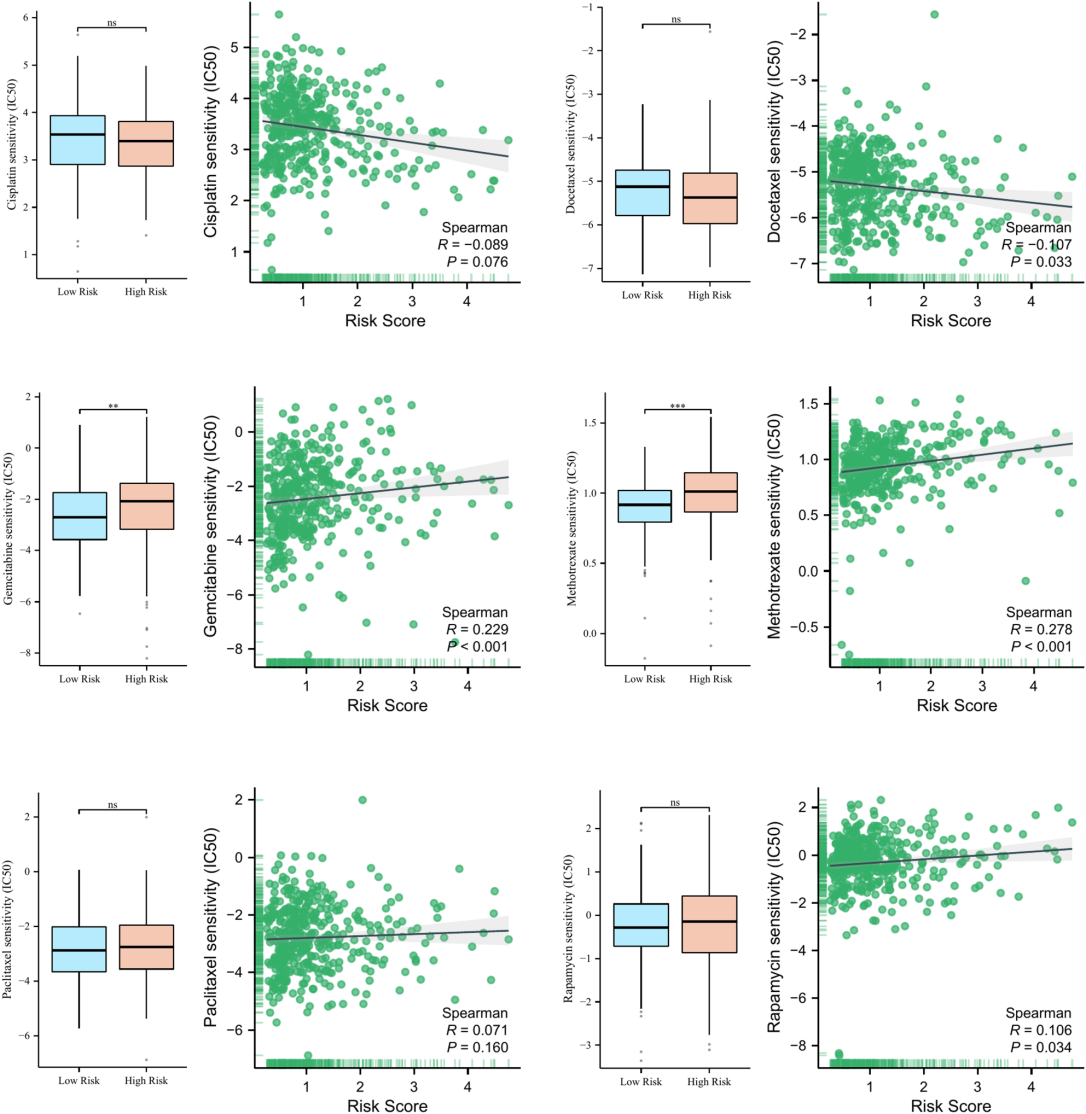
The relationship between risk scores and IC50 values of six common chemotherapy drugs. *ns* not significant; **p < 0.01; ***p < 0.001.

To explore the guiding significance of risk model in the chemotherapeutic and targeted therapy of BLCA patients, we evaluated the relationship between risk score and IC50 values of several common chemotherapy drugs (such as Cisplatin, Docetaxel, Gemcitabine, Methotrexate, Paclitaxel and Rapamycin). Figure 10 showed that patients in high-risk group may be more sensitive to Methotrexate, while patients in low-risk group may benefit more from Gemcitabine chemotherapy.

### The CTSE in risk model is closely related to the development of BLCA

**Figure 11:**
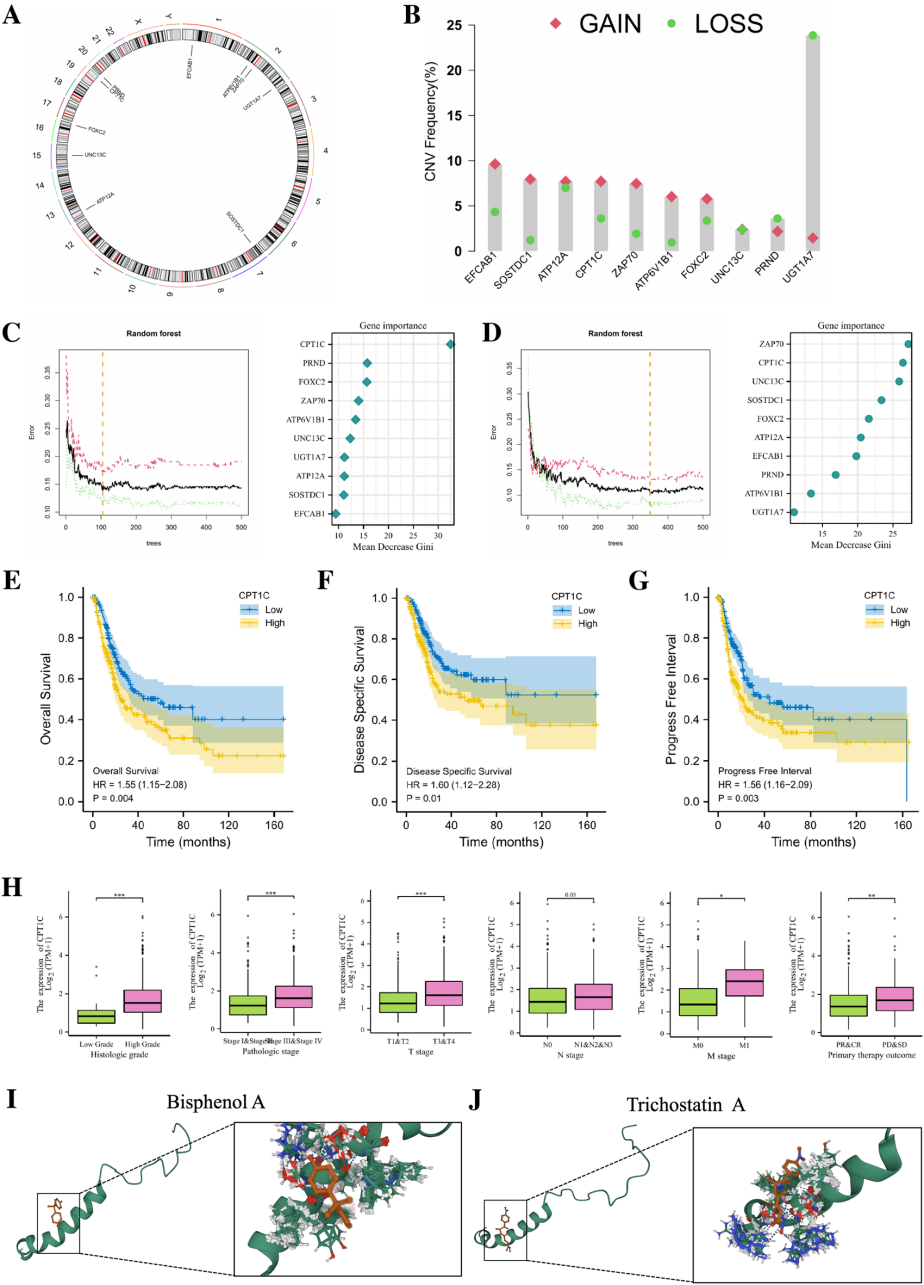
(**A**) Circus plots of chromosome distributions of selected genes from the risk model. (**B**) Frequencies of gain and loss for selected genes from the risk model. (**C**, **D**) To further evaluate the importance of these genes for the prognostic contribution of LUAD patients, we performed a random forest analysis of these genes based on risk scores and patient NMF classifications and found that the mean decrease Gini of CPT1C was higher in both analyses. (**E**, **G**) The Kaplan–Meier survival curve showing the relationship between the expression of CPT1C and overall survival (OS), disease-specific survival (DSS), and progress-free interval (PFI). (**H**) The expression of CPT1C in TCGA-BLCA patients with different histologic grade, pathologic stage, T stage, N stage, M stage and primary therapy outcomes. (**I**) The simulated binding between CPT1C and Bisphenol A. (**J**) The simulated binding between CPT1C and Trichostatin A. *p < 0.05; **p < 0.01; ***p < 0.001.

For the 10 genes in the risk model, Fig. 11A shows their respective locations on chromosomes. The analysis of copy number variation (CNV) frequency showed that the vast majority of the 10 model genes exhibited significant CNV changes (Fig. 11B). To assess the importance of the above genes for the prognosis of BLCA patients, we performed a Random Forest algorithm on the above 10 genes based on the risk score and different patient classifications, and found that the average Gini degradation of CPT1C ranked high in both analysis (Fig. 11C, D). In view of the significant impact of CPT1C on the prognosis of BLCA patients, we chose CPT1C for further exploration. Through K-M survival analysis we found that BLCA patients with high expression of CPT1C had significantly worse OS, DSS, and PFI compared to BLCA patients with low expression of CPT1C (Fig. 11E–G). The results of clinical correlation analysis indicated that BLCA patients with higher pathological grade, later clinical stage, later TNM stage, and poor treatment efficacy may have high expression of CPT1C, which further proved that the expression of CPT1C was closely related to the development of BLCA (Fig. 11H). Using the CTD database for screening, we identified two drugs (Bisphenol A and Tichostatin A) targeting CPT1C based on Autodock molecular docking and toxicology studies. Bisphenol A can tightly bind to CPT1C and down-regulate the mRNA expression of CPT1C. Its macromolecular docking simulated binding energy is − 7.64 (kcal/mol) (Fig. 11II). Tichostatin A can also tightly bind to CPT1C and down-regulate the mRNA expression of CPT1C. The simulated binding energy of its macromolecular docking is − 7.32 (kcal/mol) (Fig. 11J).

## Discussion

BLCA is one of the most common urological malignancies and has caused considerable morbidity and mortality, placing a heavy burden on the health care system^3,26^. Despite the advancements in smoking cessation, surgery, and systemic therapy, encompassing chemotherapy and immunotherapy, the overall prognosis of individuals with BLCA has not witnessed any notable improvement in survival rates during recent years^27,28^. The association between the development of BLCA and the immunological characteristics of its tumor microenvironment is significant^29,30^. Consequently, it is imperative to investigate immune-related biomarkers for prognostic prediction and treatment guidance in BLCA patients. The involvement of neutrophil extracellular traps (NETs) in facilitating the interplay between cancer progression and the tumor microenvironment is noteworthy^31^. However, there is currently a dearth of literature that identifies BLCA patients using gene expression related to NETs and constructs prognostic risk models to aid in the selection of immunotherapy and chemotherapy. Therefore, further research and development in this area are warranted.

In this study, we conducted a synthesis of NRGs by analyzing previously published articles. We then assessed the expression levels of NRGs in BLCA patients using the TCGA dataset and assigned scores to each sample accordingly. Our findings revealed a positive correlation between higher tumor malignancy and higher NETs scores. Furthermore, employing the WCGNA algorithm, we identified modular genes that exhibited strong associations with NETs scores. Leveraging these modular genes, we employed the NMF algorithm to classify all BLCA patients into two distinct subtypes, which demonstrated significant disparities in prognosis and biological characteristics. Among them, it was observed that patients in group C1 exhibited significantly improved overall survival (OS), disease-specific survival (DSS), and progression-free interval (PFI) compared to patients with BLCA in group C2. Analysis of tumor microenvironment scores revealed that patients in group C1 displayed higher stromal and immune scores, but lower tumor purity. Furthermore, immune cell infiltration analysis demonstrated a notable increase in the abundance of various immune cell infiltrates, including B cells, T cells, and macrophages, in group C1 compared to group C2. Additionally, the expression of NRGs was predominantly elevated in patients belonging to the C1 group. Moreover, IPS immunotherapy analysis indicated that patients in the C1 group may exhibit enhanced sensitivity to immunotherapy. Enrichment analysis further demonstrated notable disparities in immune-related biological processes and immunomodulation-related pathways among patients of both subtypes.

In order to investigate prognostic markers linked to NETs in BLCA, a prognostic risk model was ultimately developed employing a 10-gene signature, utilizing univariate, LASSO, and multifactorial regression analyses. Subsequently, BLCA patients were categorized into high-risk and low-risk groups based on this signature. The results of the survival analysis demonstrated that patients classified in the high-risk group exhibited significantly inferior OS rates compared to those in the low-risk group across the TCGA training set, validation set, and overall set. Among the 10 genes examined, a substantial body of literature has reported their close association with tumor development. Specifically, FOXC2 expression has been consistently linked to heightened metastatic potential and unfavorable survival outcomes in diverse solid malignancies^32^. Notably, FOXC2, functioning as a transcription factor, was observed to activate the PI3K/AKT signaling pathway and facilitate angiogenesis in glioblastoma^33^. SOSTDC1 has been identified as having a strong correlation with the development and progression of various cancer types, such as breast, kidney, gastric, and thyroid cancers^34^. Research has demonstrated that SOSTDC1 plays a role in promoting invasion of colorectal cancer and liver metastasis by interacting with ALCAM/CD166^35^. ZAP70, a cytoplasmic tyrosine kinase, is essential for initiating typical biochemical signaling pathways downstream of the TCR^36^. It has been observed that abnormal expression of ZAP70 is a common characteristic among a broad spectrum of B-cell malignancies, and the conditional expression of ZAP70 in B-cell malignancies expedites disease progression, while gene deletion impairs malignant transformation of the disease^37^. We further explored the underlying biological mechanisms responsible for the many differences between the two groups of patients at high and low risk. Enrichment analysis revealed significant activation of Hedgehog signaling, angiogenesis and epithelial-mesenchymal transition (EMT) in the tumors of patients in the high-risk group. Among them, the Hedgehog signaling pathway plays a crucial role in regulating cancer progression and drug resistance^38^. The involvement of Hedgehog signaling in the regulation of Treg differentiation and activity, as well as the transition between Tregs and Th17 cells within the tumor microenvironment, has been identified. Additionally, the processes of EMT and angiogenesis have been recognized as pivotal factors in cancer metastasis^39^. These processes collectively facilitate the departure of cancer cells from the primary tumor, invasion into neighboring tissues, dissemination to distant organs, and ultimately contribute to the progression and metastatic dissemination of malignant tumors^40,41^.

Chemotherapy and immunotherapy have emerged as prominent subjects of investigation in the context of treating patients with BLCA. To enhance the efficacy of personalized treatment for BLCA patients, we conducted a comprehensive analysis on the utility of risk models in immunotherapy. Our findings indicate that low-risk patients exhibit elevated expression levels of immune checkpoint-related genes, CD8^+^ T cell score, IPS score, and TMB in comparison to the high-risk cohort. This observation suggests that individuals in the low-risk group may display heightened responsiveness to immunotherapy. Furthermore, we also explored the potential of risk models in guiding chemotherapy for BLCA patients and found that patients in the low-risk group may benefit more from chemotherapy with gemcitabine. In order to further assess the prognostic significance of the aforementioned 10 genes in BLCA patients, our analysis utilizing the random forest algorithm revealed that CPT1C exerts a substantial influence on the survival and prognosis of BLCA patients. CPT1C, functioning as a rate-limiting enzyme in fatty acid oxidation, exhibits a close association with cancer cell viability, tumor progression, resistance to therapeutic agents, and cellular senescence^42,43^. Previous studies have documented that CPT1C-mediated fatty acid oxidation facilitates proliferation and metastasis in colorectal and gastric cancers^44–46^. In addition, recent research has demonstrated that CPT1C exerts control over the proliferation and senescence of tumor cells by regulating lipid metabolism and mitochondrial function^47^. Through the utilization of Autodock-based molecular docking and drug toxicology studies, we have successfully identified two drugs, Bisphenol A and Trichostatin A, that target CPT1C. Notably, Trichostatin A, an inhibitor of histone deacetylase, exhibits a synergistic effect with gemcitabine in the treatment of advanced BLCA, thereby enhancing its antitumor properties^48^. Consequently, future investigations may be directed towards elucidating the tumor-promoting mechanism of CPT1C in BLCA patients and evaluating the prognosis of its targeted drugs in improving the prognosis of BLCA patients.

In conclusion, a comprehensive analysis was conducted to assess the expression of NRGs in tumor tissues of BLCA patients. Subsequently, an immune-related signature was developed utilizing 10 NRGs to predict the prognosis of BLCA patients. Stratification of BLCA patients based on this signature revealed distinct prognostic characteristics, clinical features, and treatment sensitivity. The establishment of this predictive model facilitates early intervention and targeted therapy for BLCA patients, thereby potentially enhancing long-term survival rates. The findings of this study offer significant reference value for future exploration of the involvement of NETs in BLCA, as well as presenting an optimal approach for individualized treatment of BLCA patients.

### Supplementary Information

Supplementary Tables.

## Data Availability

The datasets analysed during the current study are available in the TCGA database (TCGA-BLCA) and GEO database (GSE13507 and GSE32894). Further inquiries can be directed to the corresponding author.
