# Supplementary material for: Identification of bladder cancer subtypes and predictive model for prognosis, immune features, and immunotherapy based on neutrophil extracellular trap-related genes
Source: Sci Rep. 2023 Nov 27;13:20791. doi: 10.1038/s41598-023-47824-z (PMC10682410; doi:10.1038/s41598-023-47824-z)
Supplement: Supplementary file 1 — Supplementary Tables. [file 41598_2023_47824_MOESM1_ESM.docx]

**Supplementary Table S1**: 231 neutrophil extracellular trap-related genes (NRGs).

| **Number** | **HGNC symbol** |
| --- | --- |
| 1 | **ACTB** |
| 2 | **ACTG1** |
| 3 | **ACTN1** |
| 4 | **ACTN4** |
| 5 | **AGER** |
| 6 | **AKR1B1** |
| 7 | **AKT1** |
| 8 | **AKT2** |
| 9 | **APOE** |
| 10 | **AQP9** |
| 11 | **ARPIN** |
| 12 | **ATG7** |
| 13 | **AZU1** |
| 14 | **C3** |
| 15 | **C3AR1** |
| 16 | **C5** |
| 17 | **C5AR1** |
| 18 | **CAMP** |
| 19 | **CARD11** |
| 20 | **CASP1** |
| 21 | **CASP3** |
| 22 | **CASP4** |
| 23 | **CAT** |
| 24 | **CCDC25** |
| 25 | **CCL2** |
| 26 | **CCL3** |
| 27 | **CCL4** |
| 28 | **CCL5** |
| 29 | **CD177** |
| 30 | **CD274** |
| 31 | **CD44** |
| 32 | **CEBPB** |
| 33 | **CFTR** |
| 34 | **CLCN3** |
| 35 | **CLCN4** |
| 36 | **CLCN5** |
| 37 | **CLEC4E** |
| 38 | **CLEC6A** |
| 39 | **CLEC7A** |
| 40 | **CR1** |
| 41 | **CSF2** |
| 42 | **CSF3** |
| 43 | **CTSC** |
| 44 | **CTSG** |
| 45 | **CXCL1** |
| 46 | **CXCL2** |
| 47 | **CXCL8** |
| 48 | **CXCR1** |
| 49 | **CXCR2** |
| 50 | **CXCR4** |
| 51 | **CYBA** |
| 52 | **CYBB** |
| 53 | **DDIT4** |
| 54 | **DEFA3** |
| 55 | **DNAJB1** |
| 56 | **DNASE1** |
| 57 | **ECM1** |
| 58 | **ELANE** |
| 59 | **ELN** |
| 60 | **ENO1** |
| 61 | **ENTPD4** |
| 62 | **EPHB2** |
| 63 | **F2RL2** |
| 64 | **F3** |
| 65 | **FBN1** |
| 66 | **FCAR** |
| 67 | **FCGR1A** |
| 68 | **FCGR2A** |
| 69 | **FCGR2B** |
| 70 | **FCGR3A** |
| 71 | **FCGR3B** |
| 72 | **FGA** |
| 73 | **FGB** |
| 74 | **FGG** |
| 75 | **FGL2** |
| 76 | **FN1** |
| 77 | **FPR1** |
| 78 | **FPR2** |
| 79 | **FPR3** |
| 80 | **GFI1** |
| 81 | **GP1BA** |
| 82 | **GPBAR1** |
| 83 | **GSDMD** |
| 84 | **H2AX** |
| 85 | **H3-4** |
| 86 | **HAT1** |
| 87 | **HDAC1** |
| 88 | **HDAC10** |
| 89 | **HDAC11** |
| 90 | **HDAC3** |
| 91 | **HDAC4** |
| 92 | **HDAC5** |
| 93 | **HDAC6** |
| 94 | **HDAC7** |
| 95 | **HDAC8** |
| 96 | **HDAC9** |
| 97 | **HIF1A** |
| 98 | **HMGB1** |
| 99 | **HMOX1** |
| 100 | **HRG** |
| 101 | **ICAM1** |
| 102 | **IGH** |
| 103 | **IL10** |
| 104 | **IL12A** |
| 105 | **IL17A** |
| 106 | **IL1A** |
| 107 | **IL1B** |
| 108 | **IL1RL1** |
| 109 | **IL1RN** |
| 110 | **IL33** |
| 111 | **IL36RN** |
| 112 | **IL5** |
| 113 | **IL6** |
| 114 | **ILK** |
| 115 | **IRAK4** |
| 116 | **IRF1** |
| 117 | **ITGA2B** |
| 118 | **ITGAL** |
| 119 | **ITGAM** |
| 120 | **ITGB1** |
| 121 | **ITGB2** |
| 122 | **ITGB3** |
| 123 | **JAK2** |
| 124 | **KCNN3** |
| 125 | **KLF2** |
| 126 | **KRT10** |
| 127 | **LCP1** |
| 128 | **LDLR** |
| 129 | **LPAR3** |
| 130 | **LTF** |
| 131 | **LYZ** |
| 132 | **MAP2K1** |
| 133 | **MAP2K2** |
| 134 | **MAP3K7** |
| 135 | **MAPK1** |
| 136 | **MAPK14** |
| 137 | **MAPK3** |
| 138 | **MAPK7** |
| 139 | **MCOLN3** |
| 140 | **MFN1** |
| 141 | **MFN2** |
| 142 | **MIR146A** |
| 143 | **MIR21** |
| 144 | **MIR223** |
| 145 | **MMP2** |
| 146 | **MMP9** |
| 147 | **MNDA** |
| 148 | **MPO** |
| 149 | **MTOR** |
| 150 | **MYD88** |
| 151 | **MYH9** |
| 152 | **NCF1** |
| 153 | **NCF2** |
| 154 | **NCF4** |
| 155 | **NFE2L2** |
| 156 | **NFIL3** |
| 157 | **NFKB1** |
| 158 | **NFKBIA** |
| 159 | **NLRP3** |
| 160 | **NOS2** |
| 161 | **NOX4** |
| 162 | **OPA1** |
| 163 | **ORAI1** |
| 164 | **P1BA** |
| 165 | **P2RX1** |
| 166 | **P38** |
| 167 | **PADI4** |
| 168 | **PARVB** |
| 169 | **PECAM1** |
| 170 | **PF4** |
| 171 | **PIK3CA** |
| 172 | **PIK3R1** |
| 173 | **PIK3R2** |
| 174 | **PIK3R3** |
| 175 | **PKM** |
| 176 | **PLCB** |
| 177 | **PLCG1** |
| 178 | **PLCG2** |
| 179 | **PPIF** |
| 180 | **PRKCA** |
| 181 | **PRKCB** |
| 182 | **PRKCD** |
| 183 | **PRKCG** |
| 184 | **PROCR** |
| 185 | **PRTN3** |
| 186 | **PTAFR** |
| 187 | **PTGS2** |
| 188 | **RAC1** |
| 189 | **RAC2** |
| 190 | **RAF1** |
| 191 | **RELA** |
| 192 | **RIPK1** |
| 193 | **RIPK3** |
| 194 | **S100A12** |
| 195 | **S100A8** |
| 196 | **S100A9** |
| 197 | **S1PR2** |
| 198 | **SELE** |
| 199 | **SELP** |
| 200 | **SELPLG** |
| 201 | **SGK1** |
| 202 | **SIGLEC14** |
| 203 | **SIGLEC9** |
| 204 | **SLC25A31** |
| 205 | **SLC25A4** |
| 206 | **SLC25A5** |
| 207 | **SLC25A6** |
| 208 | **SOCS3** |
| 209 | **SPP1** |
| 210 | **SRC** |
| 211 | **STAT3** |
| 212 | **SUCNR1** |
| 213 | **SYK** |
| 214 | **TGFB1** |
| 215 | **TICAM1** |
| 216 | **TIMP1** |
| 217 | **TKT** |
| 218 | **TLR2** |
| 219 | **TLR4** |
| 220 | **TLR7** |
| 221 | **TLR8** |
| 222 | **TLR9** |
| 223 | **TNC** |
| 224 | **TNF** |
| 225 | **TNFAIP3** |
| 226 | **VDAC1** |
| 227 | **VDAC2** |
| 228 | **VDAC3** |
| 229 | **VWF** |
| 230 | **WASL** |
| 231 | **XIST** |
